# An X-Linked Hyper-IgM Patient Followed Successfully for 23 Years without Hematopoietic Stem Cell Transplantation

**DOI:** 10.1155/2018/6897935

**Published:** 2018-10-14

**Authors:** Necil Kutukculer, Neslihan Edeer Karaca, Guzide Aksu, Ayca Aykut, Erhan Pariltay, Ozgur Cogulu

**Affiliations:** ^1^Ege University Faculty of Medicine, Department of Pediatric Immunology, Izmir, Turkey; ^2^Ege University Faculty of Medicine, Department of Medical Genetics, Izmir, Turkey

## Abstract

When caring for patients with life-limiting diseases, improving survival and optimizing quality of life are the primary goals. For patients with X-linked hyper-IgM syndrome (XHIGM), the treatment modality has to be decided for a particular patient regarding hematopoietic stem cell transplantation or intravenous immunoglobulin replacement therapy with* P. jiroveci* prophylaxis. A seven-year-old male patient was admitted with recurrent upper and lower respiratory tract infections and recurrent otitis media. His initial immunologic evaluation revealed low IgG and normal IgA and IgM levels with normal lymphocyte phenotyping and inadequate specific antibody responses. He was diagnosed as common variable immunodeficiency and began to receive intravenous immunoglobulin (IVIG) (0.5 gm/kg) with four-week intervals. During follow-up for 23 years under IVIG therapy, he was extremely well and never had severe infections. In 2017, targeted next generation sequencing was performed in order to understand his molecular pathology. A previously described hemizygous c.31C>T(p.Arg11Ter) mutation was found in* CD40LG* gene. The mother was heterozygous carrier for this mutation and his sister did not have any mutation. Flow cytometric analysis for* CD40LG *expression on activated T cells showed highly decreased, but not absent,* CD40LG* expression. In conclusion, diagnostic delay is a clinical problem for patients with* CD40LG* deficiency, because of low or normal IgM levels, showing that all the hypogammaglobulinemic patients, not only with high serum IgM levels, but also with normal to low IgM levels, have to be examined for* CD40LG* expression on activated T lymphocytes. Secondly, type of* CD40LG *mutations leads to enormous interpatient variations regarding serum IgM levels, CD40LG levels on activated T cells, age at diagnosis, severity of clinical findings, and follow-up therapies with or without hematopoietic stem cell therapy.

## 1. Introduction

The X-linked hyper-IgM syndrome (XHIGM, OMIM # 308230) is a rare, inherited immune deficiency disorder characterized by recurrent infections associated with low IgG and IgA and normal to increased IgM serum levels. XHIGM is caused by defects in the* CD40LG/TNFSF5* gene, encoding for* CD40 *ligand (*CD40LG*) (OMIM#300386), a molecule predominantly expressed by activated CD4+ T lymphocytes [1]. It interacts with* CD40* (OMIM#109535) molecule expressed by B lymphocytes and monocytes. Loss of interaction between them results in impairment of terminal B lymphocyte differentiation, monocyte activation, and T lymphocyte priming [1].

XHIGM patients have severe clinical features but different clinical phenotypes suggestive for combined immune deficiencies. In a report of 176 patients, 80% of patients came to medical attention due to recurrent upper and lower respiratory tract infections, 40% had varied gastrointestinal manifestations, and 11% of patients had central nervous system disease [2]. Liver disease and sclerosing cholangitis are clinical “red flags” and an important predictor of mortality in XHIGM patients similar to liver dysfunction reported to occur in a small subset of patients with common variable immunodeficiency (CVID) [1, 3].

Overall prognosis for all patients with XHIGM remains guarded, with an average of 20% survival by age 25 years [4]. In a recent retrospective study in both transplanted or nontransplanted XHIGM patients, the median postdiagnosis survival was 25 years [1]. Azzu V et al. [5] have reported that Ig replacement treated, CD40L deficient patients can live at least into the 4th decade without HSCT but that the burden of liver disease is as high as 40% in those who have not received HSCT.

In the view of the severe manifestations and outcomes associated with defects of the* CD40LG/CD40 *axis, hematopoietic stem cell transplantation (HSCT) has been successfully used to cure* CD40LG* deficiency. Since the first report in 1995 about HSCT in XHIGM, many subsequent series have been published, demonstrating improved survival and success of this therapeutic modality [6]. However, in recent years there are a few case reports showing that median survival time is similar for patients treated with or without HSCT. In this case report, we would like to present a 30-year-old XHIGM patient who has been followed up for 23 years with regular intravenous immunoglobulin and without HSCT and with no severe and opportunistic infection. In addition, we aimed to review the best treatment options to increase survival rate and quality of life for XHIGM patients.

## 2. Case Presentation

The male patient is the second child of nonconsanguineous, healthy parents and the first child is female and healthy. He was admitted to our hospital with recurrent upper and lower respiratory tract infections and recurrent otitis media when he was 7 years old. His initial immunologic evaluation revealed low IgG (307 mg/dl, normal: 1040 ± 203 mg/dl), normal IgA (112 mg/dl, normal: 108 ± 42 mg/dl), and normal to borderline IgM levels (122 mg/dl, 97 ± 42 mg/dl). He had normal numbers of CD3+ T cells (61%, 3660/mm^3^) and CD19+ B cells (20%, 1200/mm^3^) and normal numbers and percentages of other lymphocyte phenotypes (CD3+CD4+ T helper cells 29% and 1740/mm^3^, CD3+CD8+ T cytotoxic cells 28% and 1680/mm^3^, CD3-CD16+CD56+ natural killer cells 16% and 960/mm^3^). Specific IgG antibodies against tetanus and hepatitis B vaccines were both undetectable. In chest CT, he had a very mild bronchiectasis in left lower lobe of the lungs. His other laboratory tests including leukocyte, lymphocyte, haemoglobin counts, liver and kidney function tests, serologic investigations for common viruses, autoantibodies such as antinuclear antibody, direct Coombs tests, and abdominal ultrasonography were all normal. The patient did not have any sign of liver disease, there was no elevation in liver biochemistry tests such as ALT (alanine transferase), GGT (gamma-glutamyl transpeptidase), and ALP (alkaline phosphatase), and there were no findings of biliary dilatation, sclerosing cholangitis, or cirrhotic liver in ultrasound and abdominal computerized tomography.* Cryptosporidium parvum* was not detected in many stools of patient.

He was initially diagnosed as common variable immunodeficiency (CVID) and we began to give him intravenous immunoglobulin (IVIG) replacement therapy (0.5 gm/kg) with four-week intervals. During follow-up under IVIG therapy, he was extremely well and had never severe infections. He had once or twice slight pharyngitis in a year and his school attendance was excellent.

During follow-up for 22 years until last year, his IgG values just before IVIG therapies were between 500 and 600 mg/dl. IgM and IgA values were between 90 and 133 mg/dl and 70 and 104 mg/dl, respectively. When he was 20 years old, genetic analyses for* TACI (TNFRSF13B), APRIL (TNFSF13), ICOS* (inducible T cell costimulator), and* CD19* were performed and no disease causing mutations were detected in these genes known to be associated with CVID.

No more investigations were performed until 2017 and the patient still had the diagnosis of CVID and he had no severe health problems. In 22 years, he had only once easily treated bronchitis and his bronchiectatic area in the left lower lobe also recovered a few years after the diagnosis.

**Figure 1:**
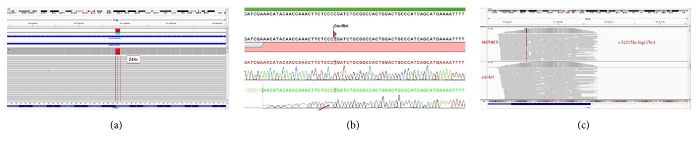
(a) Targeted next generation sequencing analysis in genomic DNA of patient. A nonsense mutation in Exon 1 (c.31C>T) changes an arginine (R) codon (CGA) into a translation termination (X) codon (TGA) at amino acid position 11 (p.Arg11Ter). (b) Sanger sequencing analysis in genomic DNA of patient. (c) The mother was heterozygous carrier and the sister had wild type gene sequences.

In 2017, we decided to perform “targeted next generation sequencing (TNGS)” in order to understand his molecular pathology. TNGS workflow based on an Ion AmpliSeq™ Primary Immune Deficiency Research Panel was designed for sequencing 264 PID genes on Ion S5™ Sequencer. A hemizygous c.31C>T(p.Arg11Ter) mutation was found in* CD40LG* gene. This mutation has been described before and found in* CD40LG* database. PCR amplification and Sanger sequencing were performed to verify the segregation of the mutation in* CD40LG* among the family members. The mother was heterozygous carrier for this mutation and his sister did not have any mutation in* CD40LG* gene (Figure 1).

Then, the patient has been examined again for flow cytometric tests and lymphocyte subsets were found to be normal again. In order to detect T cell activation and CD40LG expression, peripheral blood mononuclear cells were stimulated with phorbol 12-myristate 13-acetate (PMA, 20 ng/ml) and ionomycin (1 M) for 4 hours. The following antibody and fusion proteins were used: anti-CD40LG mAb (clone 89-76, BD biosciences, Belgium, and clone 24-31, BioLegend, CA). CD69 upregulation on CD3+ T cells stimulated with PMA and ionomycin was found to be 99.2% after stimulation while it was 1.3 before stimulation, and this finding showed us that T lmphocytes were highly activated. In CD3+ T lymphocytes gate, CD8-CD154+ cells were 16,9% after stimulation while they were 5.1% before stimulation.

This finding showed us that this mutation in* CD40LG* gene resulted in highly decreased, but not absent,* CD40LG* expression.

After XHIGM diagnosis, we did not change his treatment regimen. The patient did not accept to use subcutaneous immunoglobulin therapy, told the clinicians that his quality of life is very well on IVIG, and decided to marry in September 2018. In agreement with him, we have never thought about HSCT for this patient because of probable life-threatening risks of HSCT.

## 3. Discussion

We reported a 30-year-old male with low serum IgG and with normal serum IgA and IgM and recurrent infections before IVIG therapy. Absolute and relative numbers of lymphocyte subsets were normal, excluding the diagnosis of an X-linked agammaglobulinemia (Bruton's disease). Despite normal IgM levels, XHIGM was diagnosed after follow-up of 23 years. A literature search revealed that approximately 6.4% of X-HIGM patients had been found to have low to normal serum IgM levels [7]. Winkelstein et al. [4] reported eight cases and Gilmour et al. [8] reported four cases with low IgM, n: 8/56 and 4/17, respectively. Different studies in literature showed that many patients with low to normal serum IgM levels may not have undergone diagnostic procedures for* XHIGM* [9]. The first message for this case report has to be as follows: diagnostic delay has been noted as a clinical problem for patients with* CD40LG* deficiency, with up to 50% of patients presenting with low or normal IgM levels, so that all the hypogammaglobulinemic patients, not only with high serum IgM levels, but also with normal to low IgM levels, have to be examined for* CD40LG* expression on activated T lymphocytes.

Although most patients present with XHIGM during childhood, the age at presentation can be as late as the fifth decade of life [1]. Although our patient was admitted to Pediatric Immunology Clinic at seven years of age, the exact diagnosis could be reached after we began to perform TNGS assay when he was 29 years old. Female carriers have been reported as showing variable expression of* CD40LG* on activated T cells. As low as 32% of activated T cells expressing* CD40LG *can be seen in asymptomatic carriers [10]. Our patient's* CD40LG *expression was less than the carriers' levels.

Mutations in* CD40LG *gene are highly heterogenous.* CD40LG *database contained more than 250 public entries [11]. Besides more mutations on CD40LG were reported in other studies [9]. Considering the published results, the common type of mutations in the* CD40LG *might be missense mutations, followed by deletion/insertion mutations, nonsense mutations, and splice-site mutations [11]. Nonsense mutations like our patient's mutation in the* CD40LG* database differed between 10.5% and 26.9% in a cohort analysis of all reported different mutations [11]. Various mutations reflected the heterogeneity of not only genotypes, but also phenotypes. Our patient was receiving immunoglobulin therapy and trimethoprim/sulfamethoxazole on a continuous basis and never had severe complications, such as lung disease or opportunistic infections. We suggest that this clinical phenotype was highly related to the type of the mutation observed in our patient, because we still need further progress in better understanding of the molecular heterogeneity and clinical correlations associated with this uncommon disorder.

Azzu V et al. [5] found a higher prevalence of liver disease (33%) in XHIGM patients compared with most other studies, approximately 20% in data from a European registry and 6% in two US registries. In CVID patients, a recent study found liver disease in 5% of a cohort of 261 patients [12]. Azzu et al. [3] have reported that estimates from their cohort of over 100 adult CVID patients indicated a greater proportion of patients who have liver disease related to their primary immunodeficiency. XHIGM patients with liver disease had statistically significant elevations in liver chemistry, abnormal liver imaging including cirrhotic livers, biliary dilatation, or sclerosing cholangitis associated with* Cryptosporidium *and cytomegalovirus infection. Our patient did not have any clinical and laboratory signs of liver disease and also imaging studies including ultrasonography were all normal. On the other hand, in Azzu et al.'s study [5], crude mortality was higher in liver disease group (38%) compared with the group without liver disease (6%). Kaplan-Meier log-rank analysis showed decreased survival in XHIGM patients with liver disease [5].

The risk in the HSCT treated XHIGM patients decreased in the past two decades and those patients treated with HSCT had better scores that measured general well-being and activities of the daily life [1]. Early age at transplantation and absence of liver disease were associated with best survival [13]. Azzu et al. [5] have reported that early HSCT before age of 10 years was performed in patients without liver disease and survival was excellent. A report on HSCT for 38 XHIGM patients showed that 58% of the patients were cured, but 32% died of infection-related complications such as severe cryptosporidiosis and graft-versus-host disease [14]. From a recent report of 175 XHIGM patients collected from both US and European data, 56 of 67 subjects who underwent HSCT have survived. The median survival time was similar for transplanted and nontransplanted subjects in this cohort [2]. A report of 56 XHIGM patients in Japan showed greater overall survival rates in HSCT recipients, with significantly event-free and disease-free survival rates in patients younger than 5 years at the time of the transplant [6]. As a result, it seems that XHIGM patients who have potential HSCT donors, HSCT performed in the first decade and before the development of liver diseases, might offer not only a survival advantage, but also improved long-term general well-being. For those patients who do not have the option of HSCT, it is encouraging to see that long-term survival has improved in the last 4 decades [2].

In 2018, many questions remain. Does transplantation prevent the appearance of malignancy? How are transplant related morbidities, including graft failure and GVHD, impacting a patient's quality of life? Will gene therapy and recombinant* CD40LG* therapy be better options in the future? Long-term studies on the benefit and risk of HSCT versus immunoglobulin replacement therapy and* P.* (*carinii*)* jiroveci* prophylaxis need to be done to determine the best course of action for a particular patient.

This case report underlines the fact that* CD40LG *mutations leading to immunoglobulin class switch defects are showing enormous interpatient variations regarding serum IgM levels, CD40LG levels on activated T cells, age at diagnosis, severity of clinical findings, and follow-up therapies with or without HSCT.
